# Contrast Staining may be Associated with Intracerebral Hemorrhage but Not Functional Outcome in Acute Ischemic Stroke Patients Treated with Endovascular Thrombectomy

**DOI:** 10.14336/AD.2018.0807

**Published:** 2019-08-01

**Authors:** Hong An, Wenbo Zhao, Jianguo Wang, Joshua C Wright, Omar Elmadhoun, Di Wu, Shuyi Shang, Chuanjie Wu, Chuanhui Li, Longfei Wu, Jian Chen, Jiangang Duan, Hongqi Zhang, Haiqing Song, Yuchuan Ding, Xunming Ji

**Affiliations:** ^1^Department of Neurology, Xuanwu Hospital, Capital Medical University, Beijing, China.; ^2^China-America Institute of Neuroscience, Xuanwu Hospital, Capital Medical University, Beijing, China.; ^3^Department of Rehabilitation, Beijing Tongren Hospital, Capital Medical University, Beijing, China.; ^4^Wayne State University School of Medicine, Detroit, MI, USA.; ^5^Department of Neurosurgery, Wayne State University School of Medicine, Detroit, MI, USA.; ^6^Department of Anesthesiology, Critical Care, and Pain Medicine, Beth Israel Deaconess Medical Center/Harvard Medical School, Massachusetts, USA.; ^7^Department of Emergency, Xuanwu Hospital, Capital Medical University, Beijing, China.; ^8^Department of Neurosurgery, Xuanwu Hospital, Capital Medical University, Beijing, China

**Keywords:** post-interventional contrast staining, endovascular thrombectomy, outcome, intracerebral hemorrhage

## Abstract

To evaluate the incidence of post-interventional contrast staining (PICS) in acute ischemic stroke (AIS) Chinese patients who were treated with endovascular thrombectomy (ET) and investigate potential association of PICS with functional outcome and intracerebral hemorrhage (ICH). This observational study was based on a single-center prospective registry study. AIS patients who underwent ET from January 2013 to February 2017 were recruited into this study. All patients had dual-energy CT (DECT) scan of the head at 12 to 24 hours post-ET. The primary outcome was the incidence of PICS. Secondary outcomes were total ICH, symptomatic ICH (sICH), 3-month functional outcome, and long-term functional outcome. One hundred and eighty patients were enrolled in this study. PICS was detected in 50 patients (28%) based on the post-interventional CT scan. We first used basic statistical analyses, showing that the incidence of both total ICH (60% vs. 25%, p<0.001) and sICH (18% vs. 8%, p=0.044) were higher in patients with PICS than those without, and fewer patients achieved no disability (mRS≤1) in the PICS group compared to the control group at both 3-month and long-term follow-up (p<0.01 each). However, multivariate regression analysis further revealed that PICS only increased total (adjusted odds ratio, 7.38; 95% confidence interval 1.66 to 32.9; p=0.009) but not sICH risk. Furthermore, the logistic regression analyses did not show statistical difference in good clinical outcomes or mortality between the two groups. PICS is a common phenomenon in Chinese AIS patients. It is associated with total ICH after ET, but it seems to have no effect on functional outcome and sICH. Further large-scale studies are warranted to validate these results.

Post-interventional contrast staining (PICS) is a common phenomenon in acute ischemic stroke (AIS) patients after endovascular thrombectomy (ET) and it usually resolves within 24-48 hrs [1, 2]. However, it is still unclear whether PICS is associated with intracranial hemorrhage (ICH) or impaired functional outcomes, thus relevant research on Chinese AIS patients is needed. Some studies reported that PICS was associated with poor clinical outcomes and hemorrhagic transformation [1, 3], while others found that extravasation of contrast neither indicated ICH nor impacted functional outcomes [2, 4]. An important reason for these discrepancies may be the different computed tomography (CT) sequences used to evaluate for PICS. Conventional CT scans have limited ability to differentiate contrast material and ICH. Whereas, dual-energy CT (DECT), a relatively new technology, enables reliable differentiation between high attenuation areas related to iodine contrast material extravasation and ICH on a voxel-by-voxel basis [5].

Different from Western countries, in which AIS is more commonly caused by cardioembolism, the etiology of most AIS patients in Eastern countries, like China, is large artery atherosclerosis (LAA), and approximately half of them have intracranial artery stenosis [6, 7]. However, the incidence of PICS remains unknown, and its effect on functional outcome and ICH in Chinese AIS patients treated with ET is still unclear.

In this study, we aimed to evaluate the incidence of PICS in Chinese AIS patients treated with ET by post-operative DECT scan and investigate whether it is related to functional outcome and ICH.

## MATERIALS AND METHODS

### Study population

The Xuanwu Stroke Center program includes a prospective registry for the study of revascularization therapy (i.e. intravenous thrombolysis and endovascular treatment) for AIS. ET has been applied according to the national guidelines with the intention to start the therapy as soon as possible after symptoms onset. All potential patients were evaluated at admission by a stroke neurologist. Candidates for intravenous thrombolysis or ET underwent emergent non-contrast head CT. CT perfusion or head and neck CT angiography (CTA) was not mandatory. Twelve to 24 hours after ET, all patients underwent a follow-up head CT. For each patient, baseline characteristics, functional outcomes, complications, and follow-up outcomes were documented in the database.

For the present study, all patients treated with ET in our neurovascular unit from January 2013 to February 2017 were selected. Eligibility criteria included 1) AIS patients treated with ET using a second-generation stent retriever device; 2) availability and accessibility of information on baseline data and at least 3-month follow-up outcome.

The present study was approved by the Ethic Committee of Xuanwu Hospital of Capital Medical University. All patients or their legally authorized representative provided written informed consent at admission to our hospital.

### Date collection

We used the following variables of patients from the database: age, sex, location of occluded artery, time from onset to puncture and recanalization, initial stroke severity assessed by the National Institutes of Health Stroke Scale (NIHSS), initial CT examination evaluated by Alberta Stroke Program Early CT Score (ASPECTS) and posterior circulation ASPECTS (pc-ASPECTS), bridging therapy utilization, risk factors for cerebrovascular diseases, etiology according to the Trial of Org 10172 in Acute Stroke Treatment (TOAST), operation details, ICH, functional outcome at 3 months and long-term follow up assessed by modified Rankin Scale (mRS).

### CT imaging

DECT examination was performed for all patients at 12-24 hours after ET while a plain head CT was acquired at 72±6 hours post-treatment or whenever an ICH was indicated by clinical evidence. A 64-channel multidetector DECT equipment (Somatom definition FLASH; Siemens) was used for imaging at 90kV and 150kV respectively. A three-material decomposition algorithm was applied to differentiate normal brain parenchyma, hemorrhage, and iodine contrast through comparison with unenhanced, virtual images and iodine overlay images. Material with energy-dependent attenuation compatible with iodine was mapped on the iodine overlay image and not on virtual non-contrast (VNC) image, whereas material similar to water (ie, blood) was mapped on the virtual non-contrast image and not on the iodine overlay image [5, 8, 9]. In addition, the differentiation of contrast material extravasation and ICH was confirmed by comparing the plain CT scan at 72 hours post-treatment with the previous CT scan. Like other neuroimages, all CT images were separately analyzed by a neurologist and a neuroradiologist, and disagreement was resolved by reaching a consensus. If no consensus could be reached, another reviewer made the final decision.

### Endpoints

The primary outcome was the incidence of PICS. PICS was defined as the hyperattenuation areas only seen on the iodine overlay image but not on the virtual non-contrast image of DECT, which was confirmed by comparing plain CT scan at 72 hours post-treatment with the previous CT scan [3]. Secondary outcomes included 1) total ICH; 2) symptomatic ICH (sICH); 3) functional outcome at 3-month; and 4) functional outcome at long-term follow up (medium 12 months). Total ICH was defined according to the Heidelberg Bleeding Classification [10] and sICH was defined according to the ECASS-III study, which classified sICH as any apparent extravascular blood within the brain or cranium that was associated with clinical deterioration (an increase of 4 points or more in NIHSS) [11]. Functional outcome was assessed by modified Ranks scale (mRS), with mRS scores 0-1 indicating no disability, mRS scores 0-2 indicating functional independence, and mRS scores 6 indicating death.

**Table 1:** Demographic and clinical characteristics of all patients.

	All patients N=180	PICS N=50	Control N=130	p values
Age at onset, mean±SD, y	61.3±12.8	61.8±13.1	60.8±12.4	0.581
Male, n (%)	129 (72)	30 (60)	99 (76)	0.031*
NIHSS, median (IQR)	20 (14-30)	20 (15-33)	21 (14-29)	0.615
ASPECTS/pc-ASPECTS, median (IQR)	9 (8-10)	9 (8-10)	9 (8-10)	0.198
Treatment with IV alteplase, n (%)	42 (23)	10 (20)	32 (25)	0.512
OTP time, median (IQR), min	312 (236-378)	297 (238-372)	315 (232-379)	0.909
OTR time, median (IQR), min	379 (312-434)	403 (331-447)	372 (284-421)	0.058
**Vascular risk factors, n (%)**				
Hypertension	121 (67)	29 (58)	92 (71)	0.102
DM	48 (26)	12 (24)	36 (28)	0.616
AF	49 (27)	22 (45)	27 (21)	0.001*
Smoking	80 (44)	19 (40)	61 (48)	0.317
**Etiology of stroke, n (%)**				
LAA	121 (67)	29 (58)	92 (71)	0.676
Cardioembolism	43 (24)	16 (32)	27 (21)	0.037*
Other	16 (9)	5 (10)	11 (9)	0.547
**Operational details, n (%)**				
General anesthesia	120 (67)	34 (68)	86 (68)	0.971
Additional intra-arterial thrombolysis	24 (13)	6 (12)	18 (14)	0.744
Stenting	53 (29)	26 (32)	37 (29)	0.641
Balloon	10 (6)	2 (4)	8 (6)	0.840
Tirofiban	90 (50)	22 (44)	68 (52)	0.318
TICI=0	16 (9)	5 (10)	11 (9)	0.547
TICI=2b/3	149 (83)	41 (82)	108 (72)	0.160

PICS, Post-interventional contrast staining; SD, standard deviation; IQR, interquartile range; NIHSS, National Institutes of Health Stroke Scale; ASPECT, Alberta Stroke Program Early CT score; OTP, time from symptom onset to groin puncture; OTR, time from symptom onset to recanalization; IV, intravenous; TICI, Thrombolysis in Cerebral Infarction; LAA, Large artery atherosclerosis; DM, Diabetes mellitus; AF, Atrial fibrillation.

* A P value less than 0.05 indicates statistical significance.

### Statistical analysis

All patients were divided into two groups: PICS and non-PICS (control group) groups. Baseline characteristics and clinical outcomes were compared between the two groups. In multivariate analysis, ICH was adjusted for age, baseline NIHSS, pretreatment with antiplatelet or anticoagulation, hypertension, diabetes mellitus (DM), atrial fibrillation (AF), etiology of stroke, ASPECTS/pc-ASPECTS, time from onset to groin puncture, additional intra-arterial thrombolysis and treatment with intravenous alteplase. Functional outcomes were adjusted for age, baseline NIHSS, hypertension, DM, AF, etiology of stroke, ASPECTS/pc-ASPECTS, time from onset to groin puncture time, location of stroke (posterior or anterior circulation), symptomatic ICH (sICH), and thrombolysis in cerebral infarction (TICI) scores.

Continuous variables were described by mean±SD or median (inter quartile range, IQR). T-test for independent samples or Mann-Whitney U Tests were used to detect differences between the two groups. For binary data, frequency and percentage were used to summarize data, and between-group comparisons were performed via the Chi-square, Continuity Correction or Fisher’s exact test as appropriate. All data were analyzed using SPSS 19.0 (IBM Inc.) with the significance level of p<0.05 (two-sided).

**Table 2:** Demographic and clinical characteristics of anterior circulation stroke.

	All patients N=118	PICS N=42	Control N=76	p-values
Age at onset, mean±SD	62.2 ± 12.8	66.52 ± 11.39	59.83 ± 13.05	0.006*
Male, n (%)	77 (65)	26 (62)	51 (67)	0.570
NIHSS, median (IQR)	17 (13-21)	18.5 (14-27)	16 (13-20)	0.363
ASPECT, median (IQR)	9 (8-10)	9 (8-10)	9 (8-10)	0.037*
Treatment with IV alteplase, n (%)	37 (31%)	10 (24)	27 (36)	0.189
OTP time, median (IQR)	286 (223-346)	295 (238-372)	275.5 (197-332)	0.044*
**Vascular risk factors, n (%)**				
Hypertension	68 (58)	22 (52)	46 (61)	0.391
DM	26 (22)	9 (21)	17 (22)	0.906
AF	41 (35)	19 (46)	22 (29)	0.075
Smoking	47 (40)	15 (38)	32 (44)	0.513
**Stroke etiology, n (%)**				
LAA	68 (58)	23 (55)	45 (59)	0.640
Cardioembolism	38 (32)	14 (33)	24 (32)	0.845
Other	12 (10)	5 (12)	7 (9)	0.643
**Operation, n (%)**				
General anesthesia	68 (58)	27 (64)	41 (56)	0.394
Additional intra-arterial thrombolysis	11 (9)	3 (7)	8 (11)	0.545
Stenting	35 (30)	13 (31)	22 (29)	0.819
Balloon	6 (5)	2 (5)	4 (5)	0.906
Tirofiban	56 (48)	20 (48)	36 (47)	0.979
TICI=0	12 (10)	5 (12)	7 (9)	0.643
TICI=2b/3	97 (82)	34 (79)	63 (83)	0.606

PICS, Post-interventional contrast staining; SD, standard deviation; IQR, interquartile range; NIHSS, National Institutes of Health Stroke Scale; ASPECT, Alberta Stroke Program Early CT score; OTP, time from symptom onset to groin puncture; IV, intravenous; TICI, Thrombolysis in Cerebral Infarction; LAA, Large artery atherosclerosis; DM, Diabetes mellitus; AF, Atrial fibrillation.

* A P-value less than 0.05 indicates statistical significance.

## RESULTS

A total of 180 AIS patients treated with ET were eligible for this study, including 118 patients (65.6%) of anterior circulation stroke (ACS) and 62 patients (34.4%) of posterior circulation stroke (PCS).

### Baseline Data

The baseline characteristics of all patients are summarized in Table 1. The average age at onset was 61.3±12.8-year-old. Thirty of 50 patients (60%) in the PICS group and 99 of 130 patients (76%) in the control group were male (p=0.031). Forty-two patients (23%) were treated with intravenous alteplase before ET. Median baseline NIHSS score was 20 (14-30), median baseline ASPECTS/pc-ASPECTS was 9 (8-10), median time from onset to groin puncture and recanalization was 312 (236-378) minutes and 379 (312-434) minutes respectively. Total of 149 patients (83%) achieved good or excellent reperfusion (TICI≥2b), and 16 patients (9%) failed to achieve any recanalization (TICI=0). Vascular risk factors, etiology of stroke, and operational details are also shown in Table 1. Compared to patients without PICS, more patients with PICS had a history of AF and an etiology of cardioembolism (p<0.05 each). Demographic and clinical characteristics of the patients with ACS and PCS are summarized in the Table 2 and Table 3, respectively.

### Incidence of PICS

PICS was detected in 50 patients (28%) (Table 1): 8 of 62 PCS patients (13%), and 42 of 118 ACS patients (36%), which was significantly different (Table 2 and 3, p=0.001).

### ICH

In total, ICH was detected in 63 patients (35%). While 30 of 50 patients (60%) experienced ICH in the PICS group, 33 of 130 patients (25%) in the control group experienced ICH, which was significantly different (p<0.001). The incidence of total ICH was also significantly higher in the PICS group than the control group in patients of ACS (60% vs. 38%, p=0.026) and PCS (63% vs. 7%, p<0.001) respectively (Table 4).

**Table 3:** Demographic and clinical characteristics of posterior circulation stroke.

	All patients N=62	PICS N=8	Control N=54	P-values
Age at onset, mean±SD	59.7±12.6	66.38±9.21	58.72±12.79	0.109
Male, n(%)	52 (84)	4 (50)	48 (89)	0.023*
NIHSS, median (IQR)	28 (18-32)	33 (15.5-35.75)	26 (18-30)	0.212
ASPECT, median (IQR)	9 (8-10)	7.5 (7-8)	9 (8-10)	0.004*
Treatment with IV alteplase, n (%)	5 (8)	0	5 (9)	0.369
OTP time, median (IQR)	385 (306-445)	317 (220-373)	397 (314-460)	0.079
**Vascular risk factors, n (%)**				
Hypertension	53 (86)	7 (88)	46 (85)	0.862
DM	22 (36)	3 (38)	19 (35)	0.898
AF	8 (13)	3 (38)	5 (9)	0.026*
Smoking	33 (53)	4 (50)	29 (54)	0.845
**Stroke etiology, n (%)**				
LAA	53 (86)	6 (75)	47 (87)	0.367
Cardioembolism	5 (8)	2 (25)	3 (6)	0.059
Other	4 (6)	0	4 (7)	0.426
**Operation, n (%)**				
General anesthesia	52 (84)	7 (88)	45 (83)	0.765
Additional intra-arterial thrombolysis	13 (21)	3 (38)	10 (19)	0.218
Stenting	18 (29)	3 (38)	15 (28)	0.572
Balloon	4 (7)	0	4 (7.4)	0.426
Tirofiban	34(55)	2 (25)	32 (59)	0.069
TICI=0	4 (7)	0	4 (7)	0.427
TICI=2b/3	52 (84)	7 (88)	45 (83)	0.765

PICS, Post-interventional contrast staining; SD, standard deviation; IQR, interquartile range; NIHSS, National Institutes of Health Stroke Scale; ASPECT, Alberta Stroke Program Early CT score; OTP, time from symptom onset to groin puncture; IV, intravenous; TICI, Thrombolysis in Cerebral Infarction; LAA, Large artery atherosclerosis; DM, Diabetes mellitus; AF, Atrial fibrillation.

* A P value less than 0.05 indicates statistical significance.

Nineteen of 180 (11%) patients suffered from sICH, including 10 patients (8%) in the control group and 9 patients (18%) in the PICS group (p=0.044). In patients with PCS, 3 of 8 patients (38%) with PICS suffered from sICH compared with 1 of 54 patients (2%) without PICS (p<0.001) (Table 4).

### Three-month Functional Outcome

Forty-three of 180 patients (24%) had no disability at 3-months follow up, including 5 patients in the PICS group and 38 patients in the control group, which was significantly different (10% vs. 29%, p=0.007) (Table 4). A total of 74 out of 180 patients (41%) achieved functional independence. Sixteen of 50 patients (32%) with PICS achieved functional independence, compared to 58 of 130 patients (45%) without PICS, but no significant difference was detected (p=0.123). Forty-nine of 180 patients (27%) died at 3-month follow up, including 17 patients in the PICS group and 32 patients in the control group, which was not significant (34% vs. 25%, p=0.205). However, the mortality of PCS patients was significantly higher in the PICS group than in the control group (75% vs. 30%, p=0.012) (Table 4).

### Long-term Functional Outcome

The median length of follow up was 12 (3-26.5) months, and 161 patients finished the long-term follow up (Table 5). For those who died, the follow-up periods were recorded from stroke onset to the time that the subject died. A total of 52 out of 161 patients (32%) had no disability at long-term follow up, including 7 patients with PICS (16%) and 44 without PICS (38%), which was significantly different (p=0.006). Seventy-four of 161 patients (46%) achieved functional independence, including 16 patients with PICS (36%) and 58 patients without PICS (50%), but no significant difference was detected (p=0.099). Forty-three of 161 patients (33%) died at long-term follow up, including 18 patients with PICS (40%) and 35 patients without PICS (30%) (p=0.234).

### Multivariate regression analysis

To determine whether PICS was independently associated with ICH or clinical outcome, multivariate regression analysis was performed. The results revealed that PICS was only independently associated with total ICH (OR 7.38, 95% CI 1.66-32.90; p=0.009). However, PICS did not have an impact on sICH, 3-months functional outcome or long-term functional outcome (p>0.1 each). Additionally, there was no correlation between PICS and mortality when controlling for other factors (Table 6). All factors that were included in the multivariate analysis to predict ICH are summarized in Table 7. Only PICS and additional intra-arterial thrombolysis affected ICH significantly.

**Table 4:** Intracerebral hemorrhage and 3-month follow up.

	All patients, N=180				Anterior circulation stroke, N=118			Posterior circulation stroke, N=62		
	All	PICS N=50	Control N=130	p-values	PICS N=42	Control N=76	p-values	PICS N=8	Control N=54	p-values
Total ICH, n (%)	63 (35)	30 (60)	33 (25)	<0.001*	25 (60)	29 (38)	0.026*	5 (63)	4 (7)	<0.001*
sICH, n (%)	19 (11)	9 (18)	10 (8)	0.044*	6 (14)	9 (12)	0.703	3 (38)	1 (2)	<0.001*
3-month mRS, n (%)										
mRS, 0-1	43 (24)	5 (10)	38 (29)	0.007*	5 (12)	29 (38)	0.003*	0	9 (17)	0.212
mRS, 0-2	74 (41)	16 (32)	58 (45)	0.123	15 (36)	43 (56)	0.030	1 (13)	15 (28)	0.357
mRS, 6	49 (27)	17 (34)	32 (25)	0.205	11 (26)	16 (21)	0.525	6 (75)	16 (30)	0.012*

PICS, Post-interventional contrast staining; ICH, Intracranial Hemorrhage; sICH, symptomatic Intracranial Hemorrhage; mRS, modified Ranks Scale.

* A P value less than 0.05 indicates statistical significance.

## DISCUSSION

In this study, we found that PICS was detected in over one-fourth of AIS patients treated by ET, and it was an independent predicator for total ICH. However, PICS may not be associated with the functional outcome and sICH. Collectively, these results support the clinical relevance of DE-CT to identify early BBB disruption in patients with AIS receiving ET. These results also hint that PICS without final ICH may only require routine follow-up.

The incidence of PICS in our cases was approximately 28%, which was lower than previous studies (31.2% and 60.3%) [1, 4]. This discrepancy could be attributed to several factors. Firstly, contrast staining appears to be a transient phenomenon, thus it is more likely to be detected if the time interval between the ET and the follow-up CT is short [2]. Secondly, previous studies selected different definitions for PICS and CT sequence, which potentially conflated contrast staining and ICH [1, 4]. Thirdly, AIS caused by cardioembolism are less likely in the Chinese population than in western populations [12]. The most common cause of AIS in our subjects was LAA (67%), while only 24% patients are cardioembolism. Based on our results, LAA patients were just as likely to develop PICS as other patients (p=0.676), however cardioembolism patients were more likely to develop PICS than not (p=0.037). The higher proportion of LAA patients in this study may partially account for the lower rates of PICS than previously reported.

**Table 5:** Long-term outcome.

	All patient, N=161				Anterior circulation stroke, N=107			Posterior circulation stroke, N=54		
	All	PICS N=45	Control N=116	P values	PICS N=37	Control N=70	P values	PICS N=8	Control N=46	P values
Length of follow up, month	12 (3-26.5)	3 (3-24)	15 (3-27)	0.365	12 (3-27)	16 (3-27)	0.656	1 (1-3)	10 (1-24)	0.074
mRS, 0-1, n (%)	52 (32)	7 (16)	44 (38)	0.006*	7 (19)	33 (47)	0.004*	0	11 (24)	0.121
mRS, 0-2, n (%)	74 (46)	16 (36)	58 (50)	0.099	14 (38)	42 (60)	0.029*	2 (25)	16 (34.8)	0.588
mRS, 6, n (%)	53 (33)	18(40)	35 (30)	0.234	12 (32)	17 (24)	0.367	6 (75)	18 (39)	0.060

PICS, Post-interventional contrast staining; mRS, modified Ranks Scale.

* A P-value less than 0.05 indicates statistical significance

Interestingly, we found that PICS was more frequently detected in patients with ACS than in patients with PCS. Several studies, focusing on the neuroanatomy and physiology, may provide insight into this finding. Firstly, previous studies showed that the incidence of PICS was associated with infarct volume [2], and the infarct volume in infratentorial strokes is often smaller than that of supratentorial strokes. Secondly, previous studies have identified that the posterior circulation has a greater ischemic tolerance, and BBB disruption may be delayed in the posterior circulation after stroke onset when compared to the anterior circulation [13-17].

**Table 6:** Multivariate regression analysis of the effects of PICS on ICH and functional outcome.

Outcome	Adjusted P value	OR	95% CIs	
			Lower limit	Upper limit
Total ICH#	0.018*	7.38	1.66	32.90
sICH#	0.994	--	--	--
3-months mRS, 0-1†	0.522	0.50	0.06	4.182
3-months mRS, 0-2†	0.451	2.03	0.32	12.77
3-months mortality†	0.937	1.07	0.18	6.46
PCS mortality§	0.147	9.48	0.45	198.30
Long-term mRS, 0-1†	0.141	0.22	0.03	1.65
Long-term mRS, 0-2†	0.181	3.30	0.58	17.65
Long-term mortality†	0.122	0.72	0.11	4.61

# Adjusted for age, baseline NIHSS, pretreatment with antiplatelet or anticoagulation, hypertension, diabetes mellitus, atrial fibrillation, etiology of stroke, ASPECTS/pc-ASPECTS, time from onset to groin puncture, treatment with additional intra-arterial thrombolysis, and treatment with intravenous alteplase.

† Adjusted for age, baseline NIHSS, hypertension, diabetes mellitus, atrial fibrillation, etiology of stroke, ASPECTS/pc-ASPECTS, time from onset to groin puncture, location of stroke (posterior or anterior circulation), sICH, and thrombolysis in cerebral infarction (TICI) scores.

§ Adjusted for age, baseline NIHSS, hypertension, diabetes mellitus, atrial fibrillation, etiology of stroke, ASPECTS/pc-ASPECTS, time from onset to groin puncture, sICH, and thrombolysis in cerebral infarction (TICI) scores.

* A P value less than 0.05 indicates statistical significance.

Thirdly, PCS tend to have better collateral circulation, which is critical to rescue the ischemic penumbra. Finally, both vasoreactivity and autoregulation differ between the anterior and posterior circulations. The insensitivity to autoregulation in PCS may be a factor in limiting the amount of contrast extravasation into the parenchyma [18-20]. Our study revealed that PICS was associated with ICH, which supported the concept that both PICS and ICH are successive stages of BBB damage [21]. When the ischemic injury is limited to the endothelial cells, contrast staining may be composed solely of contrast medium. However, when injury extends to the basal lamina, contrast staining may be associated with some degree of ICH [22]. Early reperfusion is crucial to prevent damage to the BBB and avoid ICH. This is also why PICS was associated with ICH and poor clinical outcomes in previous studies of intra-arterial thrombolysis which had relatively low recanalization rates [23, 24]. This finding was further supported by our data which showed that the onset to recanalization times were longer in the PICS group compared to the control.

**Table 7:** Multivariate analysis of ICH.

Variates	OR	95% CI	P-value
Age	0.969	0.91-1.03	0.296
Hypertension	3.165	0.49-20.44	0.226
DM	0.329	0.05-2.09	0.238
AF	4.935	0.62-39.18	0.131
NIHSS	0.999	0.92-1.09	0.984
Pre-Antiplatelet/ Anticoagulation	0.342	0.06-1.88	0.218
Etiology of stroke	1.736	0.87-3.48	0.120
ASPECT/pc-ASPECTS	0.590	0.36-0.97	0.036
OTP time	0.997	0.99-1.00	0.401
Additional intra-arterial thrombolysis	7.581	1.16-49.74	0.035*
Treatment with IV alteplase	2.625	0.51-13.55	0.249
PICS	6.370	1.37-29.70	0.018*

PICS, Post-interventional contrast staining; NIHSS, National Institutes of Health Stroke Scale; ASPECT, Alberta Stroke Program Early CT score; OTP, time from symptom onset to groin puncture; IV, intravenous; DM, Diabetes mellitus; AF, Atrial fibrillation.

* A P value less than 0.05 indicates statistical significance.

Contrary to Arturo's study which demonstrated that PICS was associated with poor clinical outcomes [3], our study found that PICS did not affect functional outcomes or mortality. This discrepancy may be attributed to various stroke etiologies in different ethnicities. Compared to the west, LAA is reported to be the more likely etiology for stroke in our Chinese population [7, 25]. PICS patients have higher percentage of cardioembolism events compared to LAA. This is because the former causes emergent large vessels occlusion with poor collateral circulation, which may lead to serious damage to the BBB [26, 27]. Approximately half of the acute ischemic strokes were due to cardioembolism in Arturo's study, whereas less than one fourth were due to this etiology in our study. Additionally, according to our results, mortality in PICS group was higher than the control group in PCS patients. However, this difference was not apparent after adjusting for relative factors especially for the pc-ASPECT score. This score was traditionally known as a predictor for the infarct volume and clinical outcome, with lower scores indicating larger infarct volume and worse clinical outcomes [28, 29].

Limitations of this study include small sample size which limits the utility of our conclusion. Additionally, this was an observational study and the outcome assessment was not blinded for either the baseline characteristics or treatment. Potential variables including additional use of intra-arterial thrombolysis and various etiologies of stroke might also have confounded out study results.

### Conclusion

PICS is a common phenomenon in Chinese AIS patients treated with ET. Even though PICS is associated with high incidence of ICH, no correlation to sICH or functional outcome was evident in our study. Additional, large-scale studies are needed to confirm our results and explore their influence on postoperative therapeutic guidelines.
